# Androgen Receptor Expression Governs the Seasonal Inhibition of Testicular Development and Subsequent Recovery in *Rattus norvegicus caraco*

**DOI:** 10.3390/biology14020123

**Published:** 2025-01-24

**Authors:** Yaqi Ying, Lewen Wang, Dawei Wang, Ning Li, Ying Song, Xiaohui Liu

**Affiliations:** State Key Laboratory for Biology of Plant Diseases and Insect Pests, Institute of Plant Protection, Chinese Academy of Agricultural Sciences, Beijing 100193, China; caas_yaqiying@163.com (Y.Y.); happywwener@163.com (L.W.); wangdawei02@caas.cn (D.W.); lining@caas.cn (N.L.)

**Keywords:** *Ar*, seasonal reproduction, testicular recovery, brown rat, regulation

## Abstract

Commonly in seasonal breeding animals, testicular development is inhibited prior to Sertoli cell maturation when environmental conditions become unfavorable, with recovery occurring once conditions improve. However, the precise molecular mechanisms governing this process remain unclear. We investigated the role of androgen receptor (AR) in the seasonal regulation of testicular development in a wild population of *Rattus norvegicus caraco*, a subspecies of brown rats in Northeast China residing in high-latitude regions, known for its seasonal reproductive patterns. Our results revealed a significant increase in *Ar* mRNA expression in wild rats with small testes less than 0.2 g and body weights ranging between 80 and 100 g during the nonbreeding season. Further examinations of *Ar* expression in the testicular development of *R. n. caraco* in the laboratory under different day lengths and temperatures that simulating breeding and nonbreeding seasons suggest that the maturation of Sertoli cells depends on the upregulation of *Ar* expression around a testis weight of 0.07–0.18 g, regardless of age and conditions, synchronously accompanying the initiation of the meiotic phase. When *Ar* expression was suppressed, testicular development was impeded around the stage of Sertoli cell maturation, resulting in decreased spermatogenesis and hindered growth in testis weight. Our findings elucidate how animals control the seasonal inhibition and subsequent recovery of testicular development by regulating *Ar* expression in *R. n. caraco*.

## Simple Summary

This study explores the role of androgen receptor (AR) in regulating testicular development in *Rattus norvegicus caraco*, a subspecies of brown rats from Northeast China with seasonal breeding patterns. The research found that *Ar* expression increases during the nonbreeding season, particularly in rats with small testes and body weights between 80 and 100 g. The maturation of Sertoli cells relies on the upregulation of *Ar* expression, regardless of age or environmental conditions. When *Ar* expression was suppressed, testicular development was hindered, leading to decreased spermatogenesis and slower testis growth. These results suggest that brown rats can modulate testicular development by regulating *Ar* expression as a response to seasonal environmental changes.

## 1. Introduction

Seasonal breeding animals serve as excellent models for exploring the regulatory mechanism through which animals respond and adapt to changed environments. In seasonal breeding animals, it is common for testicular development to be inhibited when environmental conditions become unfavorable, followed by recovery when conditions become favorable. The animal testis is composed of various complex cell types that undergo changes with the testicular development [1]. However, a common observation in this process is that only spermatogonia and Sertoli cells have been preserved in the seminiferous tubules of inhibited testes. This occurs in various seasonal breeding animals, such as grey squirrels (*Sciurus carolinensis*) [2], rock hyraxes (*Procavia capensis*) [3], roe deer (*Capreolus capreolus*) [4], ground squirrels (*Citellus dauricus*) [5,6] and golden hamsters (*Mesocricetus auratus*) [7], irrespective of whether the inhibition is due to impaired testicular development in juveniles or testicular atrophy in adults. Despite this widespread observation, the molecular mechanisms behind it are complex and need to be further excavated.

Androgen receptor (AR), as a steroid hormone-activated transcription factor, regulates the expression of genes involved in various biological processes, such as the maintenance of spermatogonia populations, progression through meiosis, the integrity of the blood–testis barrier, seminiferous tubule lumen formation, spermatid adhesion, and the release of mature spermatozoa [8,9,10]. Upon binding the hormone ligands such as testosterone and dihydrotestosterone, AR translocates into the nucleus, binds to the androgen response element, and recruits coregulators to either induce target gene transcriptional activation or repression, thereby affecting cellular proliferation and differentiation in target tissues [11,12,13]. In the testes, the actions of androgen and androgen receptor are mediated primarily through the testicular somatic cells [14]. Previous studies have demonstrated that knocking out *Ar* in specific cell types within the testis leads to different effects on spermatogenesis. For instance, spermatogenesis is arrested at the primary spermatocyte stage of the first meiosis when *Ar* is selectively knocked out in Sertoli cells [15]. Knocking out *Ar* in Leydig cells causes a later arrest around the secondary spermatocyte to the round spermatid stage [16]. Additionally, knocking out *Ar* in peritubular myoid cells results in azoospermia and infertility in males [12]. These studies highlight the cell-specific contribution of AR to male gonadal development.

The brown rat (*Rattus norvegicus*) is a globally distributed rodent pest species, as well as the second-most commonly used mammalian model organism for laboratory research [17]. Our previous study showed that the subspecies *R. n. caraco* exhibits seasonal reproductive activity in Northeast China [18]. The testicular development of *R. n. caraco* is inhibited before the stage of Sertoli cell maturation in most rats with a bodyweight less than 80 g and is recovered in most rats with a bodyweight higher than 80 g in winter [19]. The maturity of Sertoli cells serves as a critical checkpoint in the process of testicular development. In inhibited testes, Sertoli cells remain immature, and the meiotic process has not started. Conversely, in the recovered testes, Sertoli cells exhibit maturity and initiate the meiotic process. Additionally, the rapid growth of testis weight correlates with the maturation of Sertoli cells. Given the critical role of the AR in regulating Sertoli cell proliferation and maturation, as well as spermatogenesis [20,21], we assumed that *Ar* might play a critical role in regulating seasonal testicular development in the wild *R. n. caraco* population.

In this study, we characterized the feature of *Ar* expression at different postnatal development stages under both natural and simulated breeding and nonbreeding conditions. We found that upregulated *Ar* expression is a prerequisite for testicular development recovery from an inhibited status.

## 2. Materials and Methods

### 2.1. Sample Collection

#### 2.1.1. Sampling of Testis Tissues in Wild *R. n. caraco* Populations

To compare the *Ar* expression of the brown rats in the breeding season and nonbreeding season, we chose testis tissues from 83 individuals (breeding season: *n* = 24; nonbreeding season: *n* = 59) that were previously collected in Harbin City, Heilongjiang Province (E126°41′, N48°14′), from 2008 to 2016 using the cage-trapping method and stored in a freezer at −80 °C [22]. Rats captured in March, May, and June were considered to be in the breeding season, with normally developed testes. Rats captured from September to December were representatives of the nonbreeding season, with inhibited testes. During the breeding season, the day length increased from 12 h 17 min to 15 h 44 min with a maximum temperature of 35 °C; during the nonbreeding season, the day length decreased from 12 h 17 min to 8 h 40 min with a minimum temperature of −30 °C.

#### 2.1.2. Sampling of Testis Tissues in Laboratory Strain of *R. n. caraco* at Different Developmental Stages

Because most wild rats were weaned when they were captured, we characterized the *Ar* expression along with development in weaned rats using a laboratory strain. This strain was domesticated from the same wild *R. n. caraco* population in Harbin City, China, in 2012 and has been maintained in the lab for over ten generations. Despite over ten generations of rearing under laboratory conditions, these brown rats remain sensitive to photoperiod and temperature. After weaning at a postnatal day (PND) of 21, the offspring were raised individually in cages at room temperature (25 ± 1.5 °C). The testis tissues from 52 rats were collected at PND21, 30, 45, 60, 75, 90, and 120 (*n* = 8, 7, 11, 9, 7, 5, and 5, respectively) in our previous study [22].

#### 2.1.3. Sampling of Testis Tissues Around the Stage of Sertoli Cell Maturation Under Simulated Breeding and Nonbreeding Conditions

Because the maturation of Sertoli cells is a main physiological checkpoint in inhibiting testicular development in the wild *R. n. caraco* population [19], we sampled testis tissues around this stage under simulated breeding and nonbreeding conditions. The conditions of a room temperature of 23 °C and 14 h of light per day (abbreviated as T23DL14) were used to simulate the breeding season, and the conditions of a low temperature of 8 °C and 10 h of light per day (abbreviated as T8DL10) were used to simulate the nonbreeding season. Adult males and females were paired for one week under the T23DL14 condition, and then the females were moved into the two different conditions. The newborn offspring were group-raised until PND23. Testis tissues were collected at PND18 (PND18-T23DL14; *n* = 13; PND18-T8DL10; *n* = 12) and PND23 (PND23-T23DL14; *n* = 16; PND23-T8DL10; *n* = 19) around period of Sertoli cell maturation.

The animal handling in this study strictly followed the guidelines for the ethical use and compassionate care of animals established by the Institute of Plant Protection, Chinese Academy of Agricultural Sciences (protocol code IPP-202009R003).

### 2.2. Hematoxylin and Eosin (H&E) Staining and Histological Analysis

**Figure 1:**
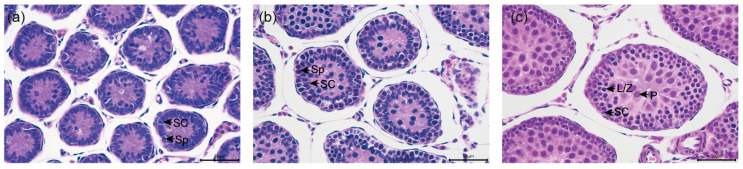
Illustration of spermatogenic status in the testes of *R. n. caraco* (**a**–**c**). Magnifications of 400 times were used in each picture. The scale bar represents 50 μm. Sp = spermatogonia; SC = Sertoli cell; L/Z = leptotene/zygotene spermatocytes; P = pachytene spermatocytes.

To assess the developmental stages of the testes, H&E staining (Soonbio, Beijing, China) of testicular sections was performed using standard procedures [23,24]. Testicular samples from all individuals in the simulated breeding and nonbreeding season were stained for H&E. For each individual, 50 seminiferous tubule sections with round or near-round appearance were randomly selected, and their diameters were measured. The average diameter was then calculated for each individual. The testicular developmental status was evaluated by examining over 100 seminiferous tubules in a single testicle slice of each rat using light microscopy and the Image Analysis System 11 application (Beijing Changheng Rongchuang Technology Co., Ltd., Beijing, China). The developmental stages of the seminiferous tubules were categorized into three phases based on the proportion of seminiferous tubules with specific cell types within each phase, including the mitotic phase, the initial phase of the meiotic phase with double-layered rosette (DLR) in tubules, and the meiotic phase [1]. An illustration of the three stages of testicular development in *R. n. caraco* is shown in Figure 1. Tubules with only spermatogonia and Sertoli cells were designated as being in the mitotic phase (Figure 1a). This phase was succeeded by the emergence of the DLRs and the onset of the meiotic phase (Figure 1b), followed by the disappearance of the DLRs and the appearance of more advanced spermatogenic cells within the tubules (Figure 1c). Finally, the percentage of each type of spermatogenic phase in all seminiferous tubules counted was counted separately for each individual. For example, meiosis phase (%): number of seminiferous tubules with the meiosis phase ÷ total number of seminiferous tubules counted × 100%; DLR phase (%): number of seminiferous tubules with double-layered rosette phase ÷ total number of seminiferous tubules counted × 100%.

### 2.3. AR Immunohistochemistry

The cell-specific expression of AR was detected using the anti-AR stain. Parasagittal paraffin sections of 4 μm thickness were prepared. The testis sections were deparaffinized with xylene and rehydrated through a series of alcohol dilutions. Subsequently, the sections were immersed in 3% hydrogen peroxide at room temperature for 10 min and treated with Tris-EDTA solution (pH = 8.0) for 3 min in a pressure cooker for antigen retrieval. After washing with PBS three times for 5 min each, the sections were incubated with the rabbit anti-AR antibody (diluted 1:500, Abcam, ab133273, Cambridge, UK) overnight at 4 °C, followed by incubation with the goat anti-rabbit antibody (diluted 1:500, ZSGB-BIO, PV-9001, Beijing, China) for 20 min at 37 °C the next day. The sections were then treated with 3,3′-diaminobenzidine (DAB) substrate to produce a dark brown precipitate, followed by hematoxylin counterstaining for 1 min at 37 °C and mounting with neutral balsam. Finally, the sections were dehydrated and mounted. For each group, at least three different individuals were selected for IHC staining and representative images were selected for presentation. PBS was used instead of primary antibody as a negative control.

### 2.4. Quantitative Real-Time PCR

The total RNA was extracted from the testis tissue of each rat using the Directzol RNA Miniprep method (ZYMO RESEARCH, Los Angeles, CA, USA). The concentration and purity of the extracted RNA were measured using a NanoDrop 2000 spectrophotometer (Thermo Fisher Scientific, Waltham, MA, USA). The RNA samples exhibited satisfactory 260/280 ratios falling within the range of 1.8 to 2.0. The integrity of the RNA was evaluated via 1% agarose gel electrophoresis, which revealed two distinct bands in appropriate proportions, indicating good RNA integrity. A precise quantity of 500 ng of total RNA was reverse-transcribed into cDNA using the Fast Quant RT Kit (TransGen Biotech, Beijing, China).

The primers for qRT-PCR were designed by Primer Premier 5.0 software based on full-length sequences of genes specific to *Rattus novergicus*. To validate the primer pairs, the amplification efficiency and specificity were checked. The forward and reverse primer sequences of the *Ar* (NM_012502.2) were F, 5′-GTACATGTGGTCAAGTGGGCCAAGG-3′, and R, 5′-GGCAAATACCATCAGTCCCATCCAGG-3′. *β-actin* (NM_031144.3) was chosen as a control gene. The forward and reverse primers for the *β-actin* were F, 5′-GGAGATTACTGCCCTGGCTCCTA-3′ and R, 5′-GACTCATCGTACTCCTGCTTGCTG-3′. The qRT-PCR was carried out by using SYBR Green PCR Master Mix (Applied Biosystems) at the following cycling conditions: 94 °C for 5 min for the initial denaturation of the cDNA hybrid; 40 cycles of 94 °C for 30 s; 60 °C for 30 s; and 72 °C for 40 s, with a final extension of 10 min at 72 °C. The relative levels of *Ar* mRNA were calculated using the 2^−ΔΔCt^ method.

### 2.5. Statistical Analysis

In wild *R. n. caraco* populations, the Kruskal–Wallis test was used to compare the differences between multiple independent subgroups in the same season, while the Mann–Whitney U test was used to examine the significance of differences between groups in different seasons. For the laboratory strain of *R. n. caraco*, one-way ANOVA was used to evaluate the testis weights and expression levels of *Ar* in testis tissue at different postnatal days. For samples from simulated breeding and nonbreeding seasons, ordinary least squares (OLS) regression analysis was used to evaluate the correlation between two physiological indicators, and the Mann–Whitney U test was used to test for differences between the two treatment conditions. Generalized linear model regression analysis using Poisson distribution was used to analyze the factors influencing the ratio of DLR-phase seminiferous tubules. These analyses were conducted using the Python package Scipy 1.0 [25] and Statsmodels 0.14.4 [26]. The hierarchical clustering was performed using the Agglomerative Clustering method in the Scikit-learn package 1.2.0 [27]. The significance level (α) was set at 0.05 for all tests. Figures were made using Seaborn and Pyplot in the Matplotlib package 3.9.2 [28] in Python Package Scipy 1.0.

## 3. Results

### 3.1. Bodyweight-Dependent Upregulation of Ar Expression in the Nonbreeding Season

**Figure 2:**
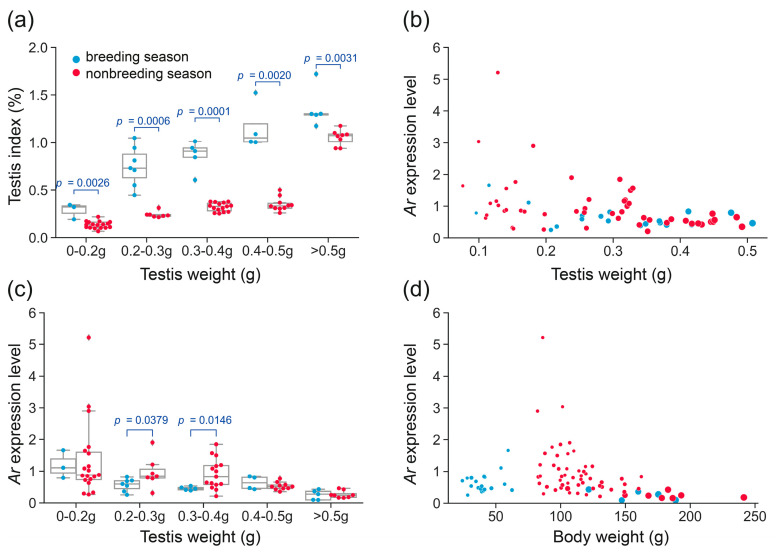
Characterization of *Ar* expression in the wild *R. n. caraco* population. (**a**) Comparison of testis index in rat testes collected between breeding and nonbreeding seasons within each rank. (**b**) Scatter plot of *Ar* expression along with testis weight growth. (**c**) Comparison of *Ar* expression in rat testes between breeding and nonbreeding seasons within each testis rank. (**d**) Scatter plot of *Ar* expression along with body weight growth of rats. The dot size is proportional to the testis weight in panels (**b**–**d**).

We analyzed the *Ar* expression in 83 rats in both the breeding and nonbreeding seasons. The rats from both seasons had a comparable range of testis weights, ranging from 0.0960 g to 2.4545 g in the breeding season and from 0.0760 g to 2.2700 g in the nonbreeding season (Supplementary Table S1). The body weights of rats with testis weights less than 0.5 g ranged from 24.2 g to 62.5 g during the breeding season and from 82.2 g to 162.3 g in the nonbreeding season (Supplementary Table S1). As the cell types within the seminiferous tubules vary with the development of testes, the testis weights were classified into five ranks by 0.1 g interval scale for comparison analysis of testis index and *Ar* expression (Figure 2a). Testis weights less than 0.2 g were grouped into one rank as only a few testes weighed less than 0.1 g.

We compared the testicular development status using the testis index between the breeding and nonbreeding seasons in each rank. The testis index represents the ratio of testis weight to body weight and is calculated using the formula (testis weight × 100)/body weight. A significantly decreased testis index was observed in rats during the nonbreeding season, suggesting an inhibited testicular development in these rats (Figure 2a), as described in previous study [22]. The scatter plot showed that the *Ar* expression was relatively higher in testes less than 0.2 g and then decreased with the growth in testis weight irrespective of the seasons (Figure 2b). Here, testis weights less than 0.2 g were predominantly found in the nonbreeding season, as most rats captured in the breeding season had developed testes weighing more than 0.2 g.

While *Ar* expression displayed a significant decline in both breeding and nonbreeding seasons with the testicular development, the variation was more pronounced in the nonbreeding season (Kruskal–Wallis test: *p* = 6.93 × 10^−5^) than in the breeding season (Kruskal–Wallis test: *p* = 0.0111). When comparing *Ar* expression in rats between the two seasons within each rank of testis weight, no significant difference was observed for testes less than 0.2 g. In contrast, for testes ranks of 0.2–0.3 g and 0.3–0.4 g, *Ar* expression was significantly higher during the nonbreeding season (Mann–Whitney test, *p* < 0.05; Figure 2c). In addition, the scatter plot analysis also revealed notably higher *Ar* expression at body weights of 80–120 g in the nonbreeding season (Figure 2d), particularly between 80 and 100 g, suggesting that *Ar* expression exhibits a bodyweight-dependent pattern during the nonbreeding season.

### 3.2. AR Expressions in Different Cell Types of Testis Around Sertoli Cell Maturation

**Figure 3:**
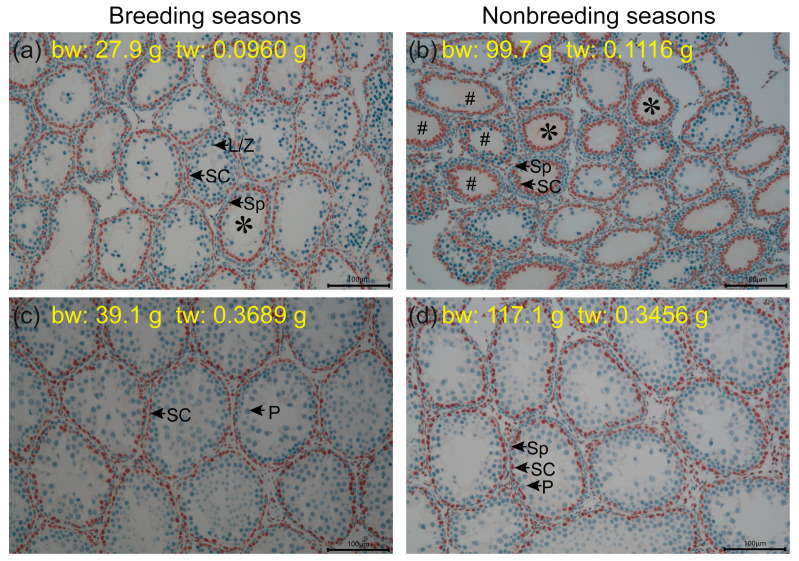
Histological features of AR immunohistochemistry of testes of four individuals collected from the wild breeding seasons and nonbreeding seasons in *R. n. caraco* (**a**–**d**). Magnifications of 200 times were used in each picture. The scale bar represents 100 μm. bw: body weight; tw: testis weight. Sp = spermatogonia; SC = Sertoli cell; L/Z = leptotene/zygotene spermatocytes; P = pachytene spermatocytes; *: double-layered rosette appearance; #: pseudostratified layer appearance.

We investigated the AR protein expression in testes using histological methods in four rats from both the breeding and nonbreeding seasons, with testes weighing greater or less than 0.2 g. IHC staining revealed that AR was predominantly expressed in Sertoli cells in all four individuals (Figure 3a–d). In the two testes, both weighing < 0.2 g, the one weighing 0.0960 g in the breeding season had already advanced to the meiotic stage, with the vast majority of Sertoli cells retreating to the basal position in the tubules (Figure 3a). In contrast, the testis weighing 0.1116 g in the nonbreeding season exhibited the appearance of more pseudostratified layers or double-layered rosettes with darker AR IHC stain, indicative of more active AR expression during Sertoli cell proliferation and maturation (Figure 3b). The histological features of the two testes weighing > 0.2 g were similar in both the breeding and nonbreeding seasons, with the appearance of pachytene spermatocytes, as shown in Figure 3c,d. While acknowledging the crucial role of AR in Sertoli cell maturation, these observations indicate that the most active AR expression occurs during Sertoli cell maturation, as shown in Figure 3b.

### 3.3. Dynamic Ar Expression Along with Testicular Development in R. n. caraco

**Figure 4:**
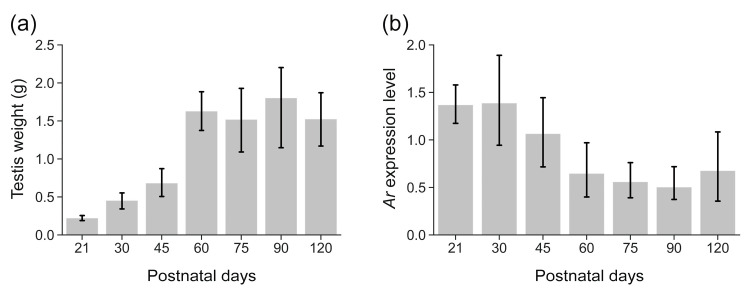
Development of testis weight (**a**) and *Ar* expression (**b**) at different postnatal testicular development stages from PND21 to PND120 in *R. n. caraco*. Data are presented as mean ± SEM.

We examined the expression of *Ar* during the development of *R. n. caraco* testes from PND23 to PND120 under the laboratory conditions of T23DL14. Consistent with our previous findings, there were significant changes in testis weights (one-way ANOVA: *p* = 1.6 × 10^−9^, Figure 4a) from PND21 to PND120 [22]. Rats weaned at PND21 had an average body weight of 33.30 ± 4.46 g and a testis weight of 0.22 ± 0.05 g, corresponding to a stage characterized by matured Sertoli cells and the presence of abundant pachytene spermatocytes. Rats at PND30 exhibited an average body weight of 50.18 ± 9.20 g and testis weight of 0.45 ± 0.15 g, representing a stage with a high number of round spermatids. The testis weight significantly increased from PND30 to PND60, reaching a maximum level that was maintained from PND60 to PND120. With the change in testis weight, the expression of *Ar* also showed significant changes from PND21 to PND120 (one-way ANOVA: *p* = 0.002, Figure 4b). *Ar* expression was at a relatively high and stable level from PND21 to PND30, followed by a notable decrease from PND30 to PND60, and was maintained at a lower level from PND60 to PND120 (Figure 4b).

### 3.4. Ar Expression Around Sertoli Cell Maturation Under Laboratory Conditions Simulating the Breeding and Nonbreeding Seasons

**Figure 5:**
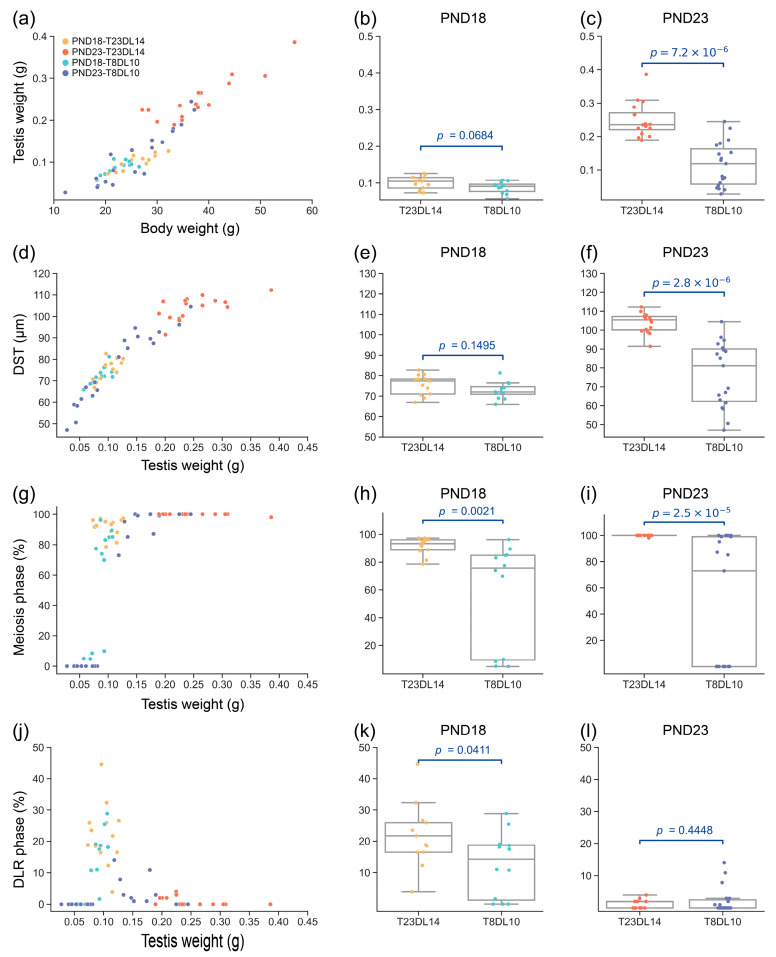
Comparison of testicular development of *R. n. caraco* at PND18 and PND23 under conditions T23DL14 and T8DL10, which simulate the breeding and nonbreeding seasons with different temperatures and photoperiods, respectively. Scatter plot of testis weight against the body weight (**a**). Comparison of testis weight between the two treatments at PND18 (**b**) and PND23 (**c**). Scatter plot of the average diameter of seminiferous tubules (DST) against the testis weight (**d**). Comparison of DST between the two treatments at PND18 (**e**) and PND23 (**f**). Scatter plot of the proportion of seminiferous tubules with meiotic phase against the testis weight (**g**). Comparison of the ratio of meiosis-phase seminiferous tubules between the two treatments at PND18 (**h**) and PND23 (**i**). Scatter plot of the proportion of seminiferous tubules in double-layered-rosette (DLR) phase against testis weight (**j**). Comparison of DLR-phase seminiferous tubules between the two treatments at PND18 (**k**) and PND23 (**l**). Legends in panels (**b**–**l**) are the same as in panel (**a**). PND: postnatal day; T23DL14: room temperature of 23 °C and 14 h of light per day; T8DL10: low temperature of 8 °C and 10 h of light per day.

We examined testicular development around Sertoli cell maturation (from PND18 to PND23) in rats treated under conditions simulating both breeding (T23DL14) and nonbreeding seasons (T8DL10). Ordinary least squares (OLS) regression analyses indicated that testis growth was significantly dependent on body weight (R^2^ = 0.877; *p* = 4.78 × 10^−28^; Figure 5a). Between the T8DL10 and T23DL14 groups, the testis weight was not significantly differentiated at PND18 (Mann–Whitney U test: *p* = 0.0684; Figure 5b), but was significantly lower in the T8DL10 group at PND23 (Mann–Whitney U test: *p* = 7.2 × 10^−6^; Figure 5c). Similarly, we found a significant linear relationship between the diameter of seminiferous tubules (DST) and testis weight (OLS regression: R^2^ = 0.881; *p* = 1.74 × 10^−28^; Figure 5d). Between the T8DL10 and T23DL14 groups, the DST was not significantly differentiated at PND18 (Mann–Whitney U test: *p* = 0.1495; Figure 5e), but was significantly decreased in theT8DL10 group at PND23 (Mann–Whitney U test: *p* = 2.8 × 10^−6^; Figure 5f).

The analysis of the testicular development phases suggested that the majority of the treated rats, except for a small proportion of rats at T8DL10, had progressed to the meiotic phase, with a testis weight exceeding 0.08 g (Figure 5g). The ratio of meiosis-phase seminiferous tubules was significantly decreased in the T8DL10 group as compared to the T23DL14 at both PND18 (Mann–Whitney U test: *p* = 0.0021; Figure 5h) and PND23 (Mann–Whitney U test: *p* = 2.5 × 10^−5^; Figure 5i). This indicates a significant inhibition of testicular development under the condition of T8DL10, which further impacted testicular development at PND23. The analysis of DLR-phase seminiferous tubules revealed a marked increase in the ratio of DLR-phase seminiferous tubules in the testes weighing from 0.0787 to 0.1795 g, exhibiting a typical Poisson distribution pattern (Figure 5j). The ratio of DLR-phase seminiferous tubules was significantly decreased in the T8DL10 group as compared to the T23DL14 group at PND18 (Mann–Whitney U test: *p* = 0.0411; Figure 5k). However, this difference disappeared at PND23 (Mann–Whitney U test: *p* = 0.4448; Figure 5l). The above results demonstrated that the inhibition of testicular development began at PND18 around Sertoli cell maturation under simulated nonbreeding season conditions. This delay subsequently resulted in declined spermatogenesis and testis weight growth and further significant differences in testis weight until the later stage of PND23.

**Figure 6:**
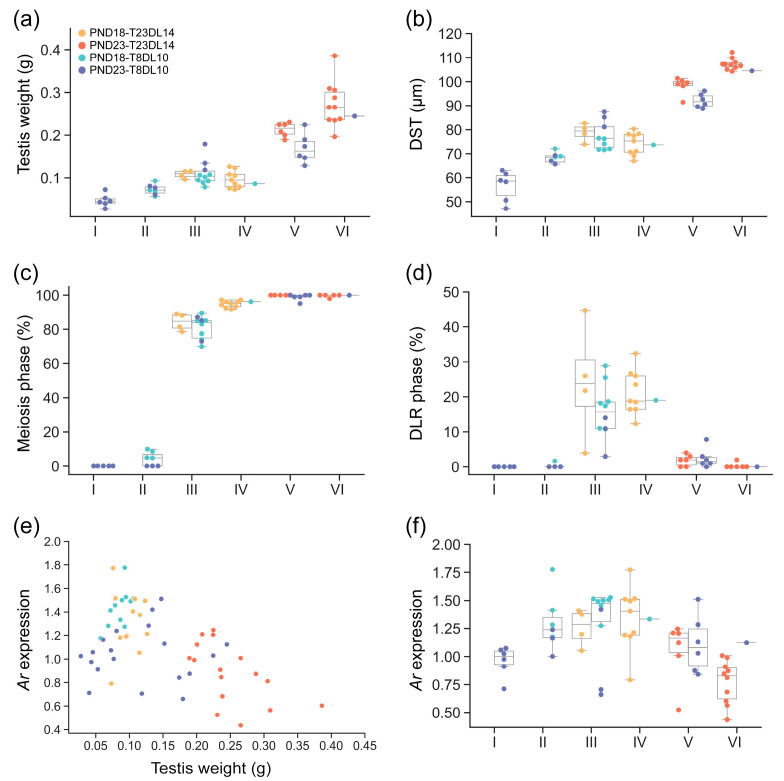
Characterization of testicular developmental parameters, *Ar* expression, and Sertoli cell maturation under T23DL14 and T8DL10. The increase in testis weight (**a**), average diameter of seminiferous tubules (DST) (**b**), and the ratio of meiosis-phase seminiferous tubules (**c**) from hierarchically clustered stage I to VI. Boxplot of the ratio of seminiferous tubules in double-layered-rosette (DLR) phase at different developmental stages of I-VI (**d**). Scatter plot of the *Ar* expression against testis weight (**e**). Boxplot of *Ar* expression at different developmental stages (**f**). Legends in panels (**b**–**f**) are the same as in panel (**a**). PND: postnatal day; T23DL14: room temperature of 23 °C and 14 h of light per day; T8DL10: low temperature of 8 °C and 10 h of light per day.

Since testicular development varied among individuals under different laboratory conditions, all testis samples at PND18 and PND23 were hierarchically clustered into six developmental stages (I–VI) based on three parameters: testis weight, DST, and the ratio of the meiotic phase. Plotting the parameters separately across the six stages revealed a consistent increase in testis weight (Figure 6a) and DST (Figure 6b) from stage I to VI. The ratio of meiosis-phase seminiferous tubules also sequentially increased from 0% to 99.82% across stages I to VI (Figure 6c), indicating a progression from early to advanced stages of development.

In contrast, the ratio of DLR-phase seminiferous tubules was relatively low in stages I-II, significantly increased in stages III-IV, and then declined in stages V-VII (Figure 6d). It was evident that the PND18 rats under T23DL14 were mainly distributed at stage III-IV, while under T8DL10 most PND18 rats were at stage III. The PND23 rats were mainly distributed at stage V-VI under T23DL14, and appeared at all stages except for stage IV, with a majority in stage I and V under T8DL10. The appearance of individuals in the slower development stages such as stage I or II under T8L10 indicated that this condition inhibited the testicular development of some individuals.

Mapping the *Ar* expression of all individuals in the four groups against testis weight reveals a relatively higher *Ar* expression around the testis weight of 0.07–0.18 g, also exhibiting a typical Poisson distribution pattern (Figure 6e). This suggested that the *Ar* expression was more related to testis weight than age or environmental conditions. The analysis of *Ar* expression across six stages reveals a gradual increase from stage I to III, peaking at stage III-IV, and then declining from stage IV to VI (Figure 6f). We noticed that the *Ar* expression is low in some PND23 individuals under T8DL10 at stage I, indicating that the suppression of *Ar* expression is critical for maintaining inhibited development status. Generalized linear model regression analyses using Poisson distribution showed that the ratio of DLR-phase seminiferous tubules, utilized as the dependent (outcome) variable, was significantly related to body weight (R^2^ = 0.7103, *p* < 0.001), testis weight (R^2^ = 0.8766, *p* < 0.001), and *Ar* expression level (R^2^ = 0.9697, *p* < 0.001). In contrast, when using the *Ar* expression as the dependent variable, no significant regression was detected between the *Ar* expression and body weight, testis weight, or DLR phase using the same model (*p* > 0.05). These results indicated that the ratio of DLR-phase seminiferous tubules depended on the *Ar* expression level, but the *Ar* expression level was not dependent on testis weight or the ratio of DLR-phase seminiferous tubules.

An obvious increase and decrease of *Ar* expression was observed from stage I to II (*p* < 0.05) and from stage VI to V (*p* < 0.05), respectively. In contrast, the ratio of the DLR-phase seminiferous tubules was not significantly differentiated between stage I and II (*p* > 0.05), or between V and VI (*p* > 0.05) (Figure 6f). The upregulation of *Ar* expression began before the emergence of the meiotic phase, and DLR appearance indicated that the emergence of DLR should be a consequence of upregulated *Ar* expression. Along with the disappearance of the DLR phase, the *Ar* expression was significantly downregulated.

In summary, the emergence of DLR in the seminiferous tubules in brown rats under simulated conditions depends on the upregulation of *Ar* expression around a testis weight of 0.07–0.18 g regardless of conditions or age. This process synchronously accompanies the emergence of the meiotic phase. The *Ar* expression remained relatively low during two stages: when the testicular development was fully inhibited, and when Sertoli cells were fully matured and meiosis was initiated entirely. 

## 4. Discussion

This study elucidated the underlying mechanism of how testicular development has been inhibited and recovered by regulating *Ar* expression in wild *R. n. caraco* during the nonbreeding season. We illustrated that the maturation of Sertoli cells depends on *Ar* expression in testes weighing between 0.07and 0.18 g, independent of conditions or age. The activation of *Ar* mRNA is an essential prerequisite for the recovery of testicular development from an inhibited status; otherwise, the testes maintain an inhibited status.

Our recent study on the reproductive seasonality of wild *R. n. caraco* revealed that 60% of juvenile rats with body weight < 80 g had a testis weight less than 0.15 g during the nonbreeding season of autumn and winter [19]. However, adult males displayed an evident recovery process with the appearance of DLRs in testicular development when an animal’s body weight reached the threshold of sexual maturity of 80 g, beginning around the testis weight of 0.15 g [19]. In this study, all of the wild rats captured in the nonbreeding season were adults with a body weight > 80 g and most had a testis weight exceeding 0.15 g, suggesting that the testes of some wild rats were in the recovery process. This study further illustrated that this recovery process in the nonbreeding season occurs around Sertoli cell maturation and is activated by the upregulation of *Ar* expression in the small testes around a similar body weight range, suggesting strong relationships among Sertoli cell maturation, *Ar* expression, and the initiation of testis recovery.

The important role of AR in the functional maturation of Sertoli cells has been demonstrated and illustrated in many previous studies [20,21]. The highest *Ar* expression is always detected around the Sertoli cell maturation along with the postnatal development of testes in many species, including rodents [29], pigs [30], monkeys [31], and humans [32,33]. The absence of AR may lead to the failure of Sertoli cell maturation and dysfunction [34,35,36]. In contrast, the elevation of AR in rat testicular Sertoli cells contributes to the improvement of Sertoli cell function, leading to the restoration of a normal seminiferous tubule structure [37]. In the wild *R. n. caraco* population and other seasonal breeders, the inhibition of testicular development and subsequent recovery occurs mainly around the stage of Sertoli cell maturation. Our findings of *Ar* expression patterns around Sertoli cell maturation indicated that this process is archived by regulating *Ar* expression.

Our present study illustrated that most seminiferous tubules had progressed to the meiotic phase along with the upregulation of *Ar* expression and the emergence of DLR, the typical marker of Sertoli cell maturation. Subsequently, the testis goes into a stage of rapid increase in weight with enhanced cell proliferation. It has been suggested that AR engages the cell cycle engine, facilitating the proliferation and survival of prostate cancer cells [38,39,40]. The overexpression of *Ar* in Sertoli cells in swine can promote the proliferation and cell cycle progression of immature Sertoli cells and accelerate the transition of Sertoli cells from the G1 phase to the S phase [41]. The absence or low expression of AR in Sertoli cells protects the testis from precocious Sertoli cell maturation in the fetal and early postnatal periods, which results in the arrest of their proliferation and the onset of pubertal spermatogenic development [42]. In addition, the expression of AR in Sertoli cells is essential for the completion of spermatogenesis [43,44]. During puberty, *Ar* expression slightly precedes AR-regulated genes, which are activated and show dynamic expression during the progression of spermatogenesis [45]. In this study, we also found that *Ar* expression was upregulated before the appearance of DLR and the meiotic phase. We speculate that animals can control testicular development by regulating *Ar* expression and sequential gene expression profiles downstream of the AR signaling pathway. The regulation of *Ar* expression should be a main checkpoint for seasonal testicular development responding to the ambient environment, which occurs around the maturation of Sertoli cells.

Previous results in the wild *R. n. caraco* population showed that the inhibition of testicular development is associated with changes in day length and temperature [19]. These were confirmed in the current study, exhibiting slower testicular development in the simulated nonbreeding season than in the simulated breeding season, as evidenced by the fact that testes in mitosis phase were all derived from T8DL10 group. Moreover, upregulated *Ar* expression and the consequent maturation of Sertoli cells occurred around testis weights of 0.07–0.18 g, and the appearance of this feature was independent of treatment conditions. This feature was identical to that of wild *R. n. caraco* populations, where the *Ar* was highly expressed in small testes (<0.2 g) in both the breeding season and nonbreeding season. Therefore, *Ar* expression is unlikely to be directly regulated by day length and temperature. The reproductive effort is physiologically regulated by allocating metabolic energy to reproduction [46], and energy restriction can delay reproductive development in male rats [47]. In brown rats, gonadal and body weights had more important impacts on sexual maturation than habitat day length and temperature [19]. Short daylength and low temperatures during the nonbreeding season influenced energy allocation states, prioritizing the safeguarding of body weight gain and survival. When body weight reaches the sexual maturity threshold, *Ar* expression and testicular developmental recovery were activated. In the nonbreeding season in the wild population, the upregulated expression of *Ar* in small testes implies a delayed and prolonged maturation of Sertoli cells in rats with body weights over 80 g. Furthermore, while higher *Ar* expression always occurs in small testes, not all small testes display higher *Ar* expression. This indicates that testicular development is still inhibited in some animals around this body weight range. We speculate that *Ar* expression is regulated mainly by energy allocation patterns along with body weight growth, although environmental changes influence this process. However, the underlying molecular mechanism of how *Ar* expression is triggered during this process is still an enigma and is worth further investigation.

## 5. Conclusions

In summary, our findings show that upregulated *Ar* expression is necessary for the developmental recovery of testes from inhibited status by promoting the maturation of Sertoli cells during stressful winter conditions in *R. n. caraco*. This process should be triggered by body-weight-dependent energy allocation to gonadal development. The suppression of *Ar* expression before Sertoli cell maturation should be a critical mechanism that maintains inhibited testicular development during winter. The regulation of *Ar* expression in Sertoli cells governs the seasonal inhibition and subsequent recovery in *R. n. caraco*.

## Data Availability

The original contributions presented in this study are included in the article. Further inquiries can be directed to the corresponding author.

## Supplementary Materials

The following supporting information can be downloaded at https://www.mdpi.com/article/10.3390/biology14020123/s1: Table S1: Physiological phenotypic data for breeding and nonbreeding seasons of Brown rat.
